# KLF5 Regulation of Exosome-Derived miR-152-3p From Bone Marrow Stem Cells Improves Ventricular Arrhythmia After Myocardial Infarction

**DOI:** 10.1155/sci/5572221

**Published:** 2025-08-09

**Authors:** Chen Wu, Xin-Yue Zou, Yi-Wen Jiang, Da-Wei Lin, Feng Jiang, Yao-Sheng Wang

**Affiliations:** ^1^Department of Cardiology, Xinhua Hospital Affiliated to Shanghai Jiao Tong University School of Medicine, Shanghai, China; ^2^Department of Cardiology, Renji Hospital Affiliated to Shanghai Jiao Tong University School of Medicine, Shanghai, China; ^3^Department of Cardiology, Zhongshan Hospital, Shanghai Institute of Cardiovascular Diseases, Fudan University, Shanghai, China; ^4^Clinical Research and Innovation Unit, Chongming Hospital Affiliated to Shanghai University of Medicine and Health Sciences, Shanghai, China; ^5^Clinical Research and Innovation Unit, Xinhua Hospital Affiliated to Shanghai Jiao Tong University School of Medicine, Shanghai, China

**Keywords:** bone marrow stem cells, cardiac fibroblasts, cardiac myofibroblasts, KLF5, miR-152-3p, myocardial infarction

## Abstract

Cardiac fibroblasts (CFs) are activated into cardiac myofibroblasts (CMFs) in myocardial infarction (MI) and promote fibrosis, playing a crucial role in deteriorating cardiac function and inducing fatal arrhythmias. Transplantation of bone marrow mesenchymal stem cells (BMSCs) has emerged as a promising therapeutic approach for ischemic heart diseases, including MI. Recent studies have indicated that BMSCs can modulate the survival, differentiation, and antifibrotic activity of CFs. Kruppel-like factor 5 (KLF5) is a significant transcription factor involved in maintaining stem cell properties. In this study, we aimed to investigate whether overexpression of KLF5 could enhance the cardioprotective characteristics of BMSCs, particularly in terms of mitigating structural and electrical remodeling. Our in vivo experiments revealed that transplantation of KLF5-overexpressing BMSCs in mice with MI led to a substantial reduction in ventricular fibrosis and the occurrence of ventricular arrhythmias (VAs). In vitro coculture experiments demonstrated that BMSCs could inhibit CFs activation and cytoskeleton protein bundling induced by hypoxia through paracrine effects, resulting in reduced expression of α-SMA and Collagen I. Furthermore, coculturing BMSCs significantly reduced the expression of connexin 43, alleviated hypoxia, increased the expression of inward-rectifier K^+^ current (Kir), and decreased voltage-dependent K^+^ (Kv) currents. Mechanistically, KLF5 enhanced the effects of BMSCs by facilitating the transfer of miR-152-3 p from BMSCs-derived exosomes to CFs. Overall, our findings show that BMSCs transplantation promotes the recovery of cardiac function and reduces the incidence of arrhythmias by inhibiting CFs activation and modulating CFs Kir current remodeling. Additionally, overexpression of KLF5 enhances the cardioprotective effects of BMSCs.

## 1. Introduction

MI, a significant global cause of mortality, often results in fatalities due to postinfarction arrhythmias and chronic heart failure [1]. As commonly understood, the differentiation of CFs into cardiac myofibroblasts (CMFs) is a critical process in the initial scar formation following MI [2, 3]. The transition of CFs to CMFs facilitates collagen synthesis and the secretion of a substantial extracellular matrix, ultimately replacing necrotic ventricular tissue with stable scar tissue [4]. Uncontrolled proliferation of CMFs can lead to fibrosis in noninfarcted remote myocardium, resulting in decreased mechanical function [5]. In the infarct border zone, CMFs establish connections with cardiomyocytes or other CMFs through gap junction proteins [6]. Due to the high membrane resistance of CMFs, the heterocellular fibroblast-cardiomyocyte coupling induces local conduction disturbances in the heart, contributing to post-MI arrhythmias. Therefore, effectively inhibiting the transformation of CFs into CMFs can enhance ventricular mechanical and electrical remodeling post-MI, subsequently reducing the incidence of arrhythmias.

BMSCs possess characteristics such as easy accessibility, rapid in vitro proliferation, multitissue differentiation potential, and low immunogenicity [7]. These attributes render BMSCs a primary source for stem cell transplantation therapy [8]. BMSCs exhibit the capacity to differentiate into cardiomyocytes, endothelial cells, or fibroblasts [9] and they secrete paracrine factors into surrounding tissues, facilitating the reversal of pathological ventricular remodeling, alleviating myocardial fibrosis, and preserving cardiac function. However, the therapeutic efficacy of transplanted cells is constrained by the ischemic microenvironment within the infarcted region, impacting cell survival. Recent studies suggest that gene modification and drug pretreatment can enhance BMSCs' survival rates, activity, and therapeutic potential [10–12]. KLF5 is a zinc finger transcription factor that governs cell signaling and is implicated in embryonic development, cardiovascular remodeling, adipogenesis, and inflammatory stress responses [13, 14]. KLF5 also plays a role in the self-renewal and maintenance of stem cells [15]. Deficiencies in KLF5 can compromise the mobilization and homing capabilities of stem cells, leading to implantation failures. Research conducted by Nakamura et al. [16] revealed that BMSCs in KLF5-deficient mice tend to differentiate preferentially into myofibroblasts. Furthermore, our prior investigations demonstrated that KLF5 targets the regulation of CFs differentiation to CMFs post-MI, indicating a significant physiological connection between BMSCs-expressed KLF5 and the transformation of CFs into CMFs during MI. These insights have prompted our exploration into whether the interplay between KLF5-mediated BMSCs and CFs is involved in the pathogenesis of MI.

The mechanisms underlying the therapeutic effects of BMSCs transplantation are multifaceted, primarily involving transdifferentiation, cell fusion, and paracrine signaling [17]. Among these, paracrine signaling is believed to be a primary mediator of their therapeutic effects, with exosomes playing a crucial role in this process [18]. Exosomes are double-membrane vesicles with diameters ranging from 40 to 160 nm, formed through the fusion of multivesicular bodies with the cell membrane [19]. These exosomes house a significant concentration of microRNAs (miRNAs), constituting over 70% of the total RNA content within these vesicles. These miRNAs exhibit distinct expression patterns in various cardiovascular diseases and play a pivotal role in regulating essential processes. MiR-152-3p is a recently identified miRNA associated with fibrosis regulation, initially recognized in the context of cancer-related disorders [20]. Xu et al. [21] have demonstrated that increased expression of miR-152-3p effectively inhibits the proliferation of CFs induced by TGF-β. Another study indicated that under hypoxic conditions, miR-152-3p can modulate CFs behaviors, including proliferation, migration, and phenotypic transition, primarily by influencing TGF-β signaling pathways [22]. Furthermore, existing research has highlighted KLF5 as a direct target of miR-152-3p [23]. This interaction leads to the binding of miR-152-3p with the BAFF promoter region, resulting in the suppression of BAFF expression within B cells. However, the precise regulatory roles and functions of these molecules in stem cells remain unclear. Further, exploration is warranted to elucidate their roles in stem cell modulation and functionality.

Our objective was to investigate the role of miR-152-3p, regulated by KLF5 in BMSCs, which may potentially aid in alleviating advanced ventricular arrhythmias (VAs) following MI by inhibiting the transition of CFs to CMFs.

## 2. Materials and Methods

### 2.1. Cell Culture

BMSCs were isolated and identified according to previously established methods [24]. In brief, 3 to 4-week-old C57BL/6 mice were utilized for BMSCs isolation. Subsequently, BMSCs were resuspended in α-minimum essential medium (α-MEM; Thermo Fisher Scientific, Waltham, MA, USA) containing 20% fetal bovine serum (FBS; Gibco, Grand Island, NY, USA) with 100 U/mL streptomycin–penicillin (Gibco), seeded into a 25 cm^2^ flask at a density of 3 × 10^6^/cm^2^, and incubated at 37°C and 5% CO_2_. The identification of BMSCs was carried out by flow cytometry analysis with cell surface markers CD29 and CD45r/b220 (Supporting Information 1: Figure S1). Cells from passages 3 to 4 were selected for subsequent experimental procedures.

CFs isolated from postnatal mice born within 24–72 h. Briefly, the hearts of neonatal mice were rapidly harvested and washed twice in cold phosphate-buffered saline (PBS). Hearts were then minced into small 0.5 to 1 mm pieces and were enzymatically digested with 0.2% pancreatin overnight. After that, the tissue was digested with 1% collagenase II (Worthington Biochemical, Lakewood, NJ) at 37°C for 30 min. After filtering and centrifuging at 800 g for 5 min, the cells were resuspended and cultured in DMEM containing 10% fetal calf serum and 100 U/mL penicillin/streptomycin for 1.5 h. Then, cell culture medium the culture medium was changed to remove unattached cardiomyocytes and replaced with fresh cell culture medium. Cells from passages 1 to 3 were used for the in vitro studies.

### 2.2. KLF5-Overexpressing BMSCs (KLF5-BMSCs) Generation

KLF5 cDNA was cloned by PCR into CMV-MCS-IRES-puromycin lentiviral vectors (Hanbio, Shanghai, China). The recombinant lentivirus was produced by cotransfecting 293T cells with PSPAX2 and PML (Hanbio, Shanghai, China). Recombinant lentiviruses were concentrated by ultracentrifugation (2 h at 50,000 *g*) 48 h after transfection from the supernatant containing lentiviruses. BMSCs were transducted with lentiviral vector at an MOI of approximately 10 in the presence of 7 μg/ml polybrene. Puromycin was added to medium at 2 mg/mL 72 h following transduction. Negative control was the empty lentivector lenti-puromycin. Stable overexpressing *KLF5*-gene cells were obtained by antibiotic selection for 3 weeks. After cell harvest, real-time PCR was used to measure KLF5 expression (Supporting Information 1: Figure S2).

### 2.3. Exosome Purification and Identification

Exosomes were isolated and purified from the supernatants of BMSCs cultures through differential centrifugation. BMSCs were cultured in DMEM supplemented with exosome-depleted serum. After a 72 h incubation, the cell-free medium was harvested and subjected to centrifugation at 2000 × *g* for 15 min at 4°C, followed by a second centrifugation at 12,000 × *g* for 45 min at 4°C. The resulting supernatants were filtered using a 0.22 μm filter (Millipore) and then underwent ultracentrifugation at 110,000 × *g* for 90 min at 4°C. The obtained pellets were washed with PBS, subjected to a second ultracentrifugation at 110,000 × *g* for 90 min at 4°C, and finally resuspended in PBS.

The identification of exosomes was further confirmed using transmission electron microscopy (TEM) and nanoparticle tracking analysis (NTA) techniques. In the TEM experiment, exosome samples were embedded in transparent resin, cut into thin sections of 60–90 nanometers, and negatively stained. These sections were then observed under TEM to examine the ultrastructure of the exosomes.

Simultaneously, NTA technology was employed to determine the size distribution and concentration of exosomes (Supporting Information 1: Figure S3). By suspending exosomes in a liquid medium containing particle-tracking agents, laser scattering technology was utilized to track and analyze the movement trajectories of exosomes, thereby determining their size. This method not only provides information on the size distribution of exosomes but also assesses their concentration, offering crucial quantitative data for further research.

### 2.4. Hypoxia Protocol

Hypoxia was induced by incubating the cells at 37°C in hypoxic incubator and adjusted 5% CO_2_ and 1% O_2_. CFs were cultured in 6-well plates for 2 days and then placed in a hypoxic incubator, where the O_2_ concentration in the chamber was maintained at 1% for 24 h. Hypoxia was terminated by removing the cells. The cells of the control were maintained in a 37°C regular culture incubator with 5% CO^2^ and 21% O_2_.

### 2.5. Coculture of CFs and BMSCs

NC-BMSCs or KLF5-BMSCs were seeded onto the lower chamber of the apparatuses (Corning, 3412) at 5 × 10^5^ cells/well, 60 μL/well (Supporting Information 1: Figure S4). The cells were incubated overnight at 37°C in 5% CO_2_. Subsequently, 3 × 10^5^ CFs were seeded onto the upper chamber. Following 24 h culture, cocultured cells underwent a 24 h hypoxia treatment. CFs were then harvested for RT-PCR and western blot analyses.

### 2.6. Transwell Migration Assay

Cell migration assays were performed using transwell culture inserts with 8 μm pore polyester membranes (Corning, 3422). CFs were seeded at a density of 5 × 10^4^ cells/well in the upper chamber in serum-free medium, while NC-BMSCs and KLF5-BMSCs were plated at 1 × 10^5^ cells/well in the lower chamber, and then both cells treated with hypoxia for 24 h. In the control group, no cell was seeded on the lower chamber. Following an incubation period of 24 h, the cells remaining on the upper chamber were removed, and the cells that had migrated through the membrane were fixed in methanol and stained with crystal violet. For each stained membrane, microscopic examination was performed, and five low-power fields (magnification × 400) were randomly selected from each chamber. All cell lines were assayed in triplicate.

### 2.7. Wound-Healing Assay

CFs were seeded on the lower chamber of the 6-well plates, while NC-BMSCs or KLF5-BMSCs were plated at 1 × 10^5^ cells/well on the upper compartment of 6-well transwell inserts (0.4 μm pore size insert, Corning, 3412), and both cells were then treated with hypoxia for 24 h. In the control group, no cell was seeded on the upper chamber. The wound-healing assay was performed by scrapping the cell monolayer of the lower chamber with a pipette tip. The gaps were observed and photographed at 0 and 24 h after scratching. The scratch area was calculated using ImageJ software based on the corresponding scratch area change rate.

### 2.8. Immunofluorescent Staining

CFs were seeded and grown on cell-climbing slices. After washed with PBS for three times, the cells were fixed with 2% paraformaldehyde for 10 min at room temperature. The slides were incubated with primary antibodies at room temperature for 2 h. Incubation with secondary antibodies was carried out at room temperature with light protection for 1 h. Rhodamine-conjugated phalloidin (F-actin; YEASEN) was used to stain the actin cytoskeleton structure. The nuclei and α-smooth muscle actin (α-SMA) expression of cells were separately identified with anti-α-SMA antibody (Abcam) and DAPI (Sigma). An Olympus Fluoview 2000 laser scanning confocal microscope was used to analyze changes in cell ultrastructure. All images were analyzed by Image-Pro Plus 6.0.

### 2.9. Western Blot

Total proteins from CFs and left ventricular tissues were extracted, and the protein concentration was measured using a BCA protein assay kit (Beyotime, P0011). Proteins were separated using 10% SDS-PAGE gel electrophoresis and were then transferred to PVDF membrane; the membrane was then blocked with tris-buffered saline (TBS)/T containing 5% nonfat dry milk and incubated with a primary antibody against α-SMA (1:3000; Abcam, Cambridge, UK), Collagen-1a (1:1000, Proteintech Group, Inc, Chicago, IL) at 4°C overnight. Blots were quantified by densitometric scanning. The level of protein expression was normalized against GAPDH controls. The assay was repeated at least three times.

### 2.10. Realtime PCR

Total RNA was isolated from fresh left ventricular tissues and CFs using EZ-press RNA Purification Kit (B0004D-100; EZBioscience, Roseville, MN, USA) according to the manufacturer's protocol. cDNA was synthesized from 2 μg RNA obtained from each sample via a reverse transcription reagent kit (11103ES70, YEASEN). Then, harvested cDNA was used for quantitative RT-PCR using SYBR Green Master Mix (11203ES03, YEASEN). The assay was repeated at least three times. All primers used in our study were as follows:

  KLF5-forward, 5′-GGCTGCGACTGGAGGTTTGC-3′
  KLF5-reverse, 5′-GGTGGTCGGAGCGGGAGAAG-3′;
  α-SMA-forward, 5′-GCGTGGCTATTCCTTCGTGACTAC-3′;
  α-SMA-reverse, 5′-CGTCAGGCAGTTCGTAGCTCTTC-3′
  Collagen I-forward, 5′-TAAGGGTCCCCAATGGTGAGA-3′;
  Collagen I-reverse, 5′-GGGTCCCTCGACTCCTACAT-3′,
  Collagen III-forward, 5′-AGTGGGAGGAATGGGTGGCTATC-3′;
  Collagen III-reverse, 5′-CTCTCCAGGTCGTCCAGGTCTTC-3′,
  GAPDH-forward, 5′-CAAGATCATTGCTCCTCCTG-3′;
  GAPDH-reverse, 5′-TCATCGTACTCCTGCTTGCT-3′,
  KCNJ2-forward, 5′-TGTGTTTTGGTTGATAGCCCTG-3′;
  KCNJ2-reverse, 5′-GCCTACGATTGACTGGAATACC-3′,
  KCNJ4-forward, 5′-GACCCTCCTCGGACCTTAC-3′;
  KCNJ4-reverse, 5′-TGCTCAGGTTGGCGAAGTAG-3′,
  KCNJ11-forward, 5′-GACGGGCTCACAGACACAC-3′;
  KCNJ11-reverse, 5′-GCTGCTCGTGCTGCTCAA-3′,
  Kv1.2-forward, 5′-GCACCCACAAGACACCTATGA-3′;
  Kv1.2-reverse, 5′-GTCTCTGGGAACTGGGCTAAG-3′,
  Kv4.2-forward, 5′-GAGCCTTTGTCACACTCCGA-3′;
  Kv4.2-reverse, 5′-CTGCTCGTTGGTTTTGGTGG-3′,
  U6-S, 5′-CTCGCTTCGGCAGCACA-3′;
  U6-A, 5′-AACGCTTCACGAATTTGCGT-3′;
  mmu-miR-152-3p, 5′-UCAGUCAUGACAGAACUUGG-3′;
  universal primer-A, 5′-TGGTGTCGTGGAGTCG-3′.

### 2.11. Flow Cytometry Analysis

When the confluency reached 80%–90%, adherent cells (within 1 × 10^6^ cells) were lightly trypsinized and collected into the centrifuge tube, and then incubated with primary antibodies for 20 min at room temperature and protected against light as well. The following monoclonal antibodies used for BMSCs were as follows: FITC-labeled anti-CD29 and FITC-labeled anti-CD45r/b220 (BD Biosciences). After incubation, cells were washed twice with PBS and then analyzed using a FACSVerse flow cytometer (BD Biosciences).

### 2.12. Dual-Luciferase Assay

Dual-Luciferase assay was employed to directly investigate the transcriptional regulation of Mir152 by KLF5. Mir152 promoter containing wild type and mutant forms were cloned into pGL3 vectors separately by Genomeditech, with the sequences provided in the Supporting Information 2. HEK 293T cells were cotransfected with individual plasmids, including pGL3-Mir152, pGL3-Mir152-MT1, and KLF5, utilizing Lipofectamine 3000. For 48 h posttransfection, cell lysates were collected and analyzed using Dual-Luciferase Assay kit, following the manufacturer's protocol. Firefly luciferase (FL) activity was normalized to Renilla luciferase (RL) relative light units and expressed as FL/RL activity ratios. Each experiment was conducted in triplicate wells to obtain average values, and three independent biological replicates were performed.

### 2.13. Animals

Adult C57BL/6 mice were supplied by the animal center of the Xinhua Hospital Affiliated to Shanghai Jiaotong University School of Medicine, Shanghai, China. Mice are house in animal care facility of our institution, 3–5 mice per cage and keep at suitable temperature with a standard 12 h light/dark cycle. All experiment were performed according to the Guide for the Care and Use of Laboratory Animals (NIH Publication number 85–23, revised 1996) and approved by the Institutional Animal Care and Use Committee of Shanghai Jiaotong University of Medicine. To avoid the effect of sex, we only choose male mice.

### 2.14. Establishment of MI Model and Cell Transplantation

The model of MI was built by ligation of the left coronary artery. In brief, mice were anesthetized with 2% isoflurane, and a 6–0 nylon suture was used to tie the left coronary artery 2–3 mm from the left atrium. After that, close the chest with 4–0 suture, immediately. Sham group mice subjected to the same procedure but without coronary artery ligature. Electrocardiogram (ECG) was recorded during the process. Successful ligature was verified by the change of ECG. The surgical intervention of each mouse requires 5–10 min.

For cell transplantation, 1 × 10^6^ NC-BMSCs or KLF5-BMSCs were suspended in 1.5 mL serum-free medium. At 1 week after MI, NC-BMSCs, KLF5-BMSCs, or serum-free medium were injected into mice via the tail vein. Within 3 weeks of BMSCs transplantation, the left ventricular internal diameter at end diastole (LVIDd), left ventricular internal dimension diastoles (LVIDs), Interventricular septal thickness at end diastole (IVSd), Interventricular septal thickness at end systoles (IVSs), ejection fraction (EF), fractional shortening (FS) was assessed using color echocardiography (Vevo 1100 system, VisualSonics, Canada).

### 2.15. Remote ECG Monitoring

This study employed an implantable electronic cardiovascular device (IECD) system, provided by Shanghai Jianlis Biotechnology Co., Ltd. The system consists of a compact electronic module (volume <5 mL, weight 15 g), two ECG monitoring electrodes, and a bipolar stimulation electrode. The external system comprises a signal transceiver, which connects to the internet or a third-generation communication network, and a personal computer equipped with software for ECG monitoring and electrical stimulation. The personal computer functions as the central workstation of the system, receiving remote digital signals and converting them into real-time ECG data.

Four weeks MI, the IECD was surgically implanted in mice and positioned near the monitoring electrodes within the laboratory setting. Remote ECG monitoring was performed by another member of the research team through an internet-based remote system operated via the personal computer. The system was utilized to monitor the timing and heart rates of premature ventricular contractions (PVCs), ventricular bigeminy (VB), ventricular trigeminy (VT1), and ventricular tachycardia (VT2) across the MI group, the NC-BMSCs transplantation group, the KLF5-BMSCs transplantation group, and the sham surgery group. In accordance with the manufacturer's specifications, the wireless signal strength of the IECD was assessed using the signal-to-noise ratio (SNR), with values of SNR > 80 dB deemed reliable.

### 2.16. Histopathology and Immunohistochemistry

We anesthetized mice with sodium pentobarbital (100 mg/kg) 4 weeks after surgery, then quickly removed and fixed the hearts in 10% formalin. Heart tissues were embedded in paraffin and cut into 4 µm-thick sections. Ventricular sections were stained with hematoxylin and eosin (HE) and Masson's trichrome (MT) stains following standard procedure. For immunohistochemistry, the sections were stained with primary antibody against collagen I (1:500, Abcam, ab6308) at 4°C overnight. Then, they were incubated with goat anti-mouse (1:500, Abcam, ab97023) for 1 h. Five different fields at ×200 magnification were randomly chosen and photographed with an inverted phase inverted microscope. The infarct area, degree of cardiac fibrosis and deposition of collagen in hearts were measured by Image-Pro Plus software.

### 2.17. Statistical Analysis

In the following analysis, data is expressed as mean ± standard deviation (SD) or percentage, and the analysis is performed using SPSS and GraphPad Prism 8. Normality of data was tested for by Shapiro–Wilk test and were considered normally distributed when the *p* value was >0.05. For data found not to be normally distributed or sample sizes were small (*N* < 6), the nonparametric alternative was used. Two-group only comparisons were performed by unpaired Student *t* test. Comparisons of three groups or more were analyzed using one-way or two-way ANOVA followed by the Bonferroni post hoc correction for multiple comparisons when appropriate. *p*  < 0.05 was considered statistically significant.

## 3. Results

### 3.1. Overexpression of KLF5 in BMSCs Significantly Potentiated the Antifibrotic Efficacy in Mice

**Figure 1:**
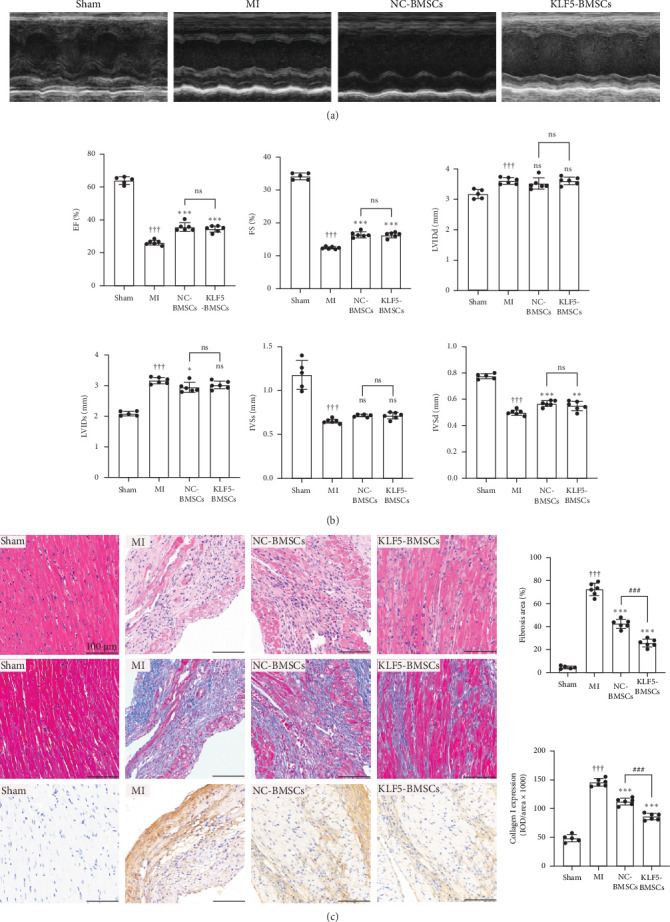
Histological and quantitative analyses of myocardial remodeling after transplantation of KLF5-BMSCs in mice. (A) Echocardiography was performed 4 weeks after MI surgery, with recording of the respective images in each group. (B) Calculation and statistical analysis of LVEF, LVFS, IVSs, IVSd, LVIDs, and LVIDd based on the echocardiography data. (C) Representative images of HE staining, Masson, and immunohistochemical staining for Collagen I. Quantitative analysis of myocardial fibrosis area and collagen deposition after MI. (^†††^*p* < 0.001 as compared with Sham group (*N* = 5); *⁣*^*∗*^*p* < 0.05, *⁣*^*∗∗*^*p* < 0.01, *⁣*^*∗∗∗*^*p* < 0.001, as compared to the MI group (*N* = 6); ^#^*p* < 0.05, ^##^*p* < 0.01, ^###^*p* < 0.001, as compared between NC-BMSCs (*N* = 6) and KLF5-BMSCs (*N* = 6); ns, non-statistically significant).

To determine the effect of KLF5 on BMSCs transplantation for the treatment of MI, a MI model was established. Subsequently, the mice received myocardial transplantation of either NC-BMSCs or KLF5-BMSCs in a randomized manner. After 4 weeks, we assessed the degree of fibrosis and collagen deposition in the infarcted area of the heart using HE staining, Masson's staining, and immunohistochemical staining. As shown in Figure 1A, 4 weeks after MI, there was a significant increase in ventricular granulation tissue, and collagen deposition and fibrosis were markedly increased in the infarcted area and its border zone. However, these effects were alleviated after BMSCs transplantation, and KLF5 overexpression significantly enhanced this effect. These results suggest that pAd-KLF5-transduced BMSCs can further reduce ventricular fibrosis after MI.

Additionally, we monitored the cardiac function of mice. After MI, cardiac function significantly declined, as evidenced by reduced left ventricular ejection fraction (LVEF), left ventricular fractional shortening (LVFS), decreased thickness of the interventricular septum at end-systole (IVS), decreased thickness of the interventricular septum at end-diastole (IVSd), and increased left ventricular internal diameter at end-diastole (LVIDd) and end-systole (LVIDs). Both NC-BMSCs and KLF5-BMSCs treatments significantly improved LVEF and LVFS compared to the MI group. However, there was no significant difference between NC-BMSCs and KLF5-BMSCs (Table 1, Figure 1B,C), indicating that pAd-KLF5-transduced BMSCs does not enhance the improvement in heart function.

### 3.2. Overexpression of KLF5 in BMSCs Reduces VAs After MI in Mice

**Figure 2:**
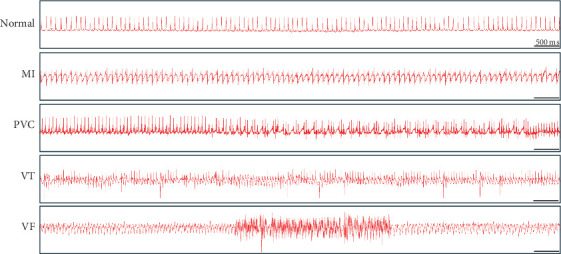
The ECG wave of normal mice, MI mice, and VA after MI were indicated.

We investigated the effect of KLF5-BMSCs transplantation on post-MI arrhythmias. To evaluate the incidence and severity of VAs, we utilized a small animal ECG monitoring device after 28 days of surgery (Figure 2). The criteria for scoring are presented in Table 2. The results showed that the transplantation of KLF5-BMSCs led to a significant reduction in the occurrence time of PVCs, VB, (VT1), and ventricular tachycardia (VT2) (Table 3), and its score was significantly lower than that of MI mice and NC-BMSCs transplantation mice (Table 4). There was no significant difference in heart rate among the MI, NC-BMSCs transplantation, KLF5-BMSCs transplantation, and sham groups.

### 3.3. KLF5 Overexpression in BMSCs Suppresses the Mechanical Properties During CFs/CMFs Transdifferentiation

**Figure 3:**
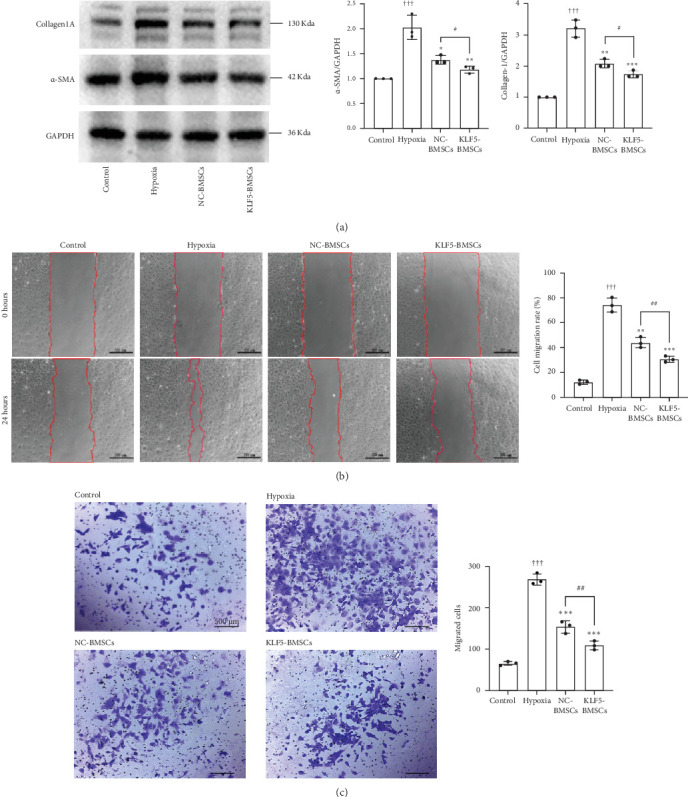
KLF5-BMSCs inhibit the transformation of CFs/CMs. (A) Western blot assay measured the expression level of α-SMA and Collagen I in CFs of each group. Grayscale analysis was quantified by Image J software. (B) Cell migration of each group was detected by a scratch assay, with the migration area of each group evaluated. (C) Transwell migration assay showed cell migration in each group. (^†††^*p* < 0.001 as compared with Control group (*N* = 3); *⁣*^*∗*^*p* < 0.05, *⁣*^*∗∗*^*p* < 0.01, *⁣*^*∗∗∗*^*p* < 0.001 as compared with Hypoxia group (*N* = 3); ^#^*p* < 0.05, ^##^*p* < 0.01, ^###^*p* < 0.001 as compared between NC-BMSCs and KLF5-BMSCs group (*N* = 3)).

Following hypoxia intervention, the protein expression levels of α-SMA and Collagen I in CFs demonstrated a more than 2-fold and 3-fold increase, respectively (*p* < 0.05) compared to the Control group. When cocultured with BMSCs, a significant reduction in the protein expression levels of α-SMA and Collagen I was observed, as compared to the hypoxia-only group. The inhibitory effect of KLF5-BMSCs coculture on the increase of α-SMA (*p* < 0.05, NC-BMSCs: 1.377 ± 0.879 vs., KLF5-BMSCs: 1.180 ± 0.069, *N* = 6) and Collagen I (*p* < 0.05, NC-BMSCs: 2.086 ± 0.131 vs., KLF5-BMSCs: 1.740 ± 0.109 *N* = 6) protein expression levels induced by hypoxia was the most significant. As shown in Figure 3A, the RT-PCR results were consistent with the western Blot results, and coculture with BMSCs could alleviate the increase of α-SMA and Collagen I RNA expression levels induced by hypoxia in CFs, with KLF5-BMSCs exhibiting a more pronounced inhibitory effect.

Wounding healing assay (Figure 3B) revealed a decrease in migratory ability of CFs after coculture with NC-BMSCs or KLF5-BMSCs followed by hypoxia intervention compared to noncocultured CFs (Control vs. Hypoxia: 12.07 ± 1.617 vs. 74.2 ± 5.742, *p* < 0.05), while KLF5-BMSCs significantly reduced the migratory ability of CFs through paracrine effects (NC-BMSCs vs. KLF5-BMSCs: 43.99 ± 4.201 vs. 30.52 ± 2.46, *p* < 0.05). Similarly, the results of the Transwell assay (Figure 3C) demonstrated that hypoxia stimulated cell migration. Coculture with KLF5-BMSCs or NC-BMSCs cells significantly inhibited the migration ability of CFs. Furthermore, the overexpression of KLF5-BMSCs notably suppressed CFs migration.

**Figure 4:**
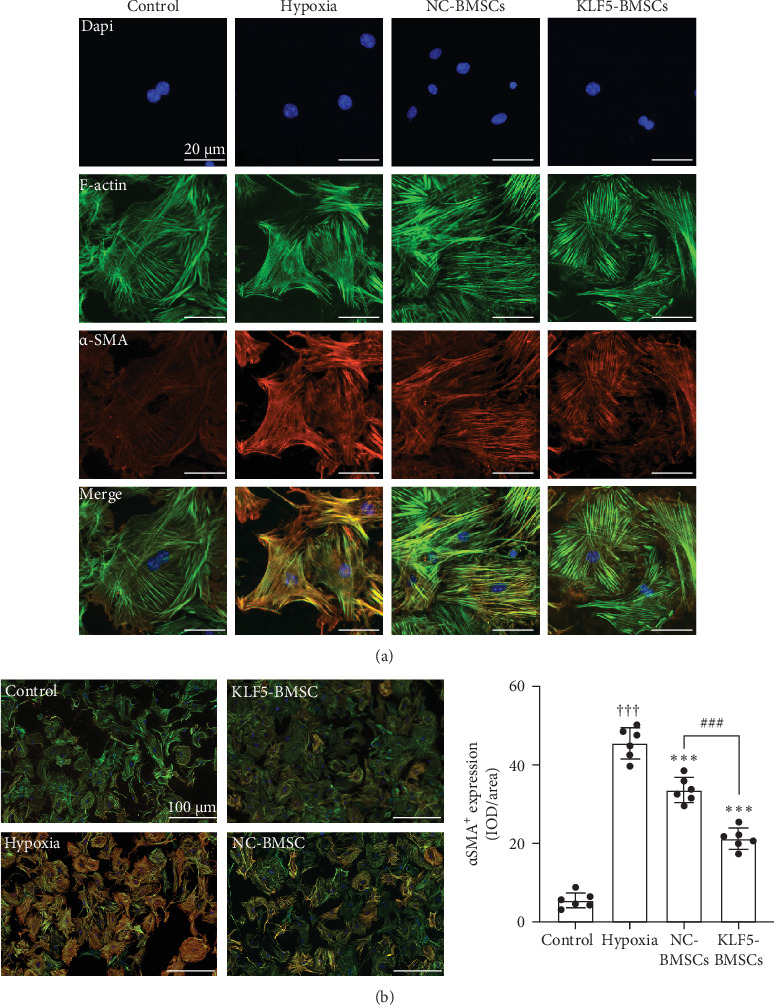
The fiber formation and assembly of CFs viewed by confocal microscope. (A) Confocal microscopic analysis depicting the formation and assembly of α-SMA^+^. F-actin, with rhodamine phalloidin (red), α-SMA, with FITC (green) and DAPI (blue). (B) The number of α-SMA^+^ cells was quantitatively evaluated. (^†††^*p* < 0.001, as compared with Control group (*N* = 6); *⁣*^*∗∗∗*^*p* < 0.001, as compared with Hypoxia group (*N* = 6); ^###^*p* < 0.001, as compared between NC-BMSCs and KLF5-BMSCs group (*N* = 6).).

Detailed ultrastructural analysis of α-SMA stress fiber formation, assembly and interaction with cytoplasmic stress fibers (F-actin) were performed using triple immunofluorescence staining. Confocal imaging showed significant differences in α-SMA^+^ cells between hypoxia intervention group and control, NC-BMSCs and KLF5-BMSCs groups. α-SMA^+^ cells from the hypoxia group easily formed α-SMA stress fibers and parallel bundles of actin filaments. In addition, the aligned α-SMA bundles in the hypoxia group were parallel to F-actin bundles and connected at their ends, allowing CFs to gain stronger contractile ability. In contrast, α-SMA^+^ cells in the control, NC-BMSCs and KLF5-BMSCs groups also contained some α-SMA stress fibers, which formed a mesh-like or bundled actin filament network but were not aligned or bundled. Similarly, the F-actin fiber networks in these groups had a mesh-like structure, with fiber bundles pointing in different directions, and were less likely to generate tension (Figure 4).

### 3.4. KLF5 Overexpression in BMSCs Suppresses the Electrical Properties During CFs/CMFs Transdifferentiation

**Figure 5:**
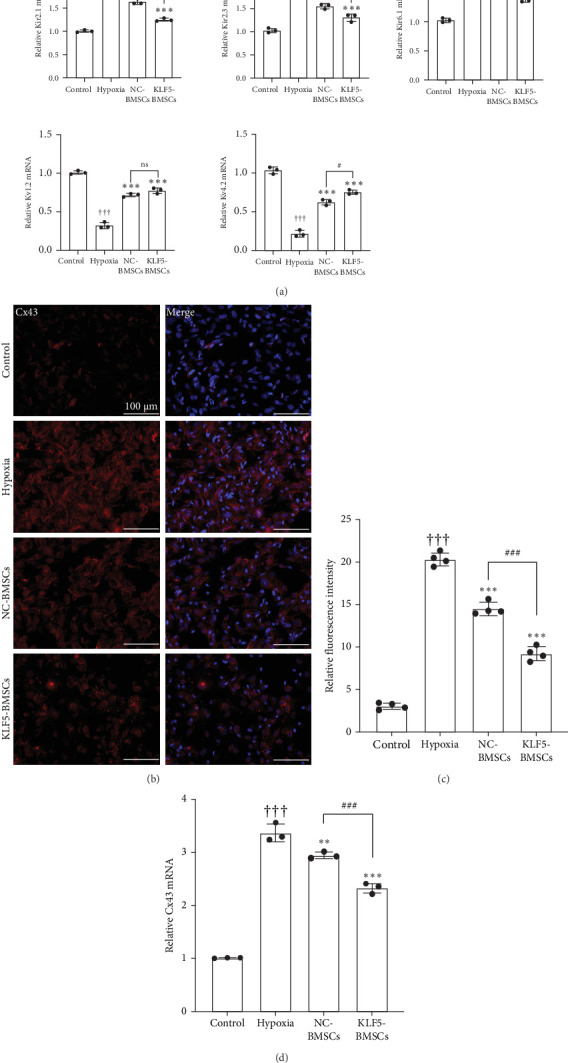
KLF5-BMSCs improve the electrical remodeling during CFs/CMFs transformation. (A) Gene expression levels of Kir2.1, Kir2.3, Kir6.1, Kv1.2 and Kv4.2 by RT-PCR in each group. (B) Immunofluorescent staining for Cx43. (C) Fluorescence intensity was quantified by using ImageJ software. (D) Gene expression levels of Cx43 by RT-PCR in each group. (^†††^*p* < 0.001, as compared with Control group; *⁣*^*∗*^*p* < 0.05, *⁣*^*∗∗*^*p* < 0.01, *⁣*^*∗∗∗*^*p* < 0.001, as compared with Hypoxia group; ^#^*p* < 0.05, ^##^*p* < 0.01, ^###^*p* < 0.001, as compared between NC-BMSCs and KLF5-BMSCs group; ns, non-statistically significant.).

As shown in Figure 5A, Kv1.2 (Control vs. Hypoxia: 1.009 ± 0.002 vs. 0.322 ± 0.04, *p* < 0.001) and Kv4.2 (Control vs. Hypoxia: 1.035 ± 0.043 vs. 0.217 ± 0.042, *p* < 0.001) in CFs was significantly downregulated, while the expression of Kir2.1 (Control vs. Hypoxia: 1.006 ± 0.025 vs. 2.065 ± 0.096, *p* < 0.001), Kir2.3 (Control vs. Hypoxia: 1.027 ± 0.046 vs. 2.103 ± 0.168, *p* < 0.001), and Kir6.1 (Control vs. Hypoxia: 1.026 ± 0.038 vs. 1.783 ± 0.025, *p* < 0.001) was significantly up-regulated. Coculturing with BMSCs could inhibit hypoxia-induced downregulation of Kv1.2 (Hypoxia vs. NC-BMSCs vs. KLF5-BMSCs: 0.322 ± 0.04 vs. 0.717 ± 0.022 vs. 0.774 ± 0.037), Kv4.2 (Hypoxia vs. NC-BMSCs vs. KLF5-BMSCs: 0.217 ± 0.042 vs. 0.624 ± 0.036 vs. 0.753 ± 0.027) expression and upregulation of Kir2.1 (Hypoxia vs. NC-BMSCs vs. KLF5-BMSCs: 2.065 ± 0.096 vs. 1.637 ± 0.057 vs. 1.249 ± 0.035), Kir2.3 (Hypoxia vs. NC-BMSCs vs. KLF5-BMSCs: 2.103 ± 0.168 vs. 1.548 ± 0.055 vs. 1.303 ± 0.079), and Kir6.1 (Hypoxia vs. NC-BMSCs vs. KLF5-BMSCs: 1.783 ± 0.025 vs. 1.602 ± 0.064 vs. 1.391 ± 0.055). Among them, the regulatory effect of KLF5 overexpression BMSCs coculture on Kv4.2 (*p* < 0.001), Kir2.1 (*p* < 0.05), and Kir6.1 (*p* < 0.001) was more significant (Table 5). In the KLF5-BMSCs coculture group, the upregulation of Kv1.2 and the downregulation of Kir2.3 were more obvious than in the NC-BMSCs group, but there was no statistical significance.

Subsequently, we detected the expression of Cx43 in different groups of CFs by cell immunofluorescence. As shown in Figure 5B, the expression of Cx43 was significantly increased in CFs after hypoxic intervention but was significantly decreased after coculture with NC-BMSCs and KLF5-BMSCs, with a more pronounced effect observed in the KLF5-BMSCs group. We then performed RT-PCR experiments on CFs in each group, and the trend was consistent with the immunofluorescence results. Cx43 expression was upregulated in the hypoxia group, and the inhibitory effect of KLF5-BMSCs on Cx43 was the most significant.

### 3.5. KLF5 Induces the Enrichment of miR-152-3p in Extracellular Vesicles

**Figure 6:**
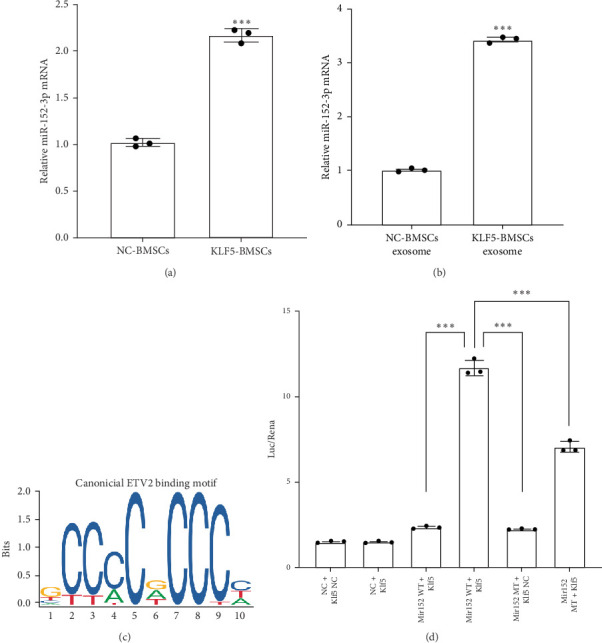
KLF5 increases the expression of miR-152-3p in BMSCs and its exosomes. (A) Relative expression levels of miR-152-3p in BMSCs transfected with empty vector or KLF5 lentivirus. (B) Relative expression levels of miR-152-3p in exosomes of BMSCs transfected with empty vector or KLF5 lentivirus. (C) Schematic of putative KLF5 binding elements on Mir152 promoter region. (D) Dual luciferase reporter gene assay of KLF5 and Mir152. (*⁣*^*∗∗∗*^*p* < 0.001 as compared with NC-BMSCs group (*N* = 3).).

**Figure 7:**
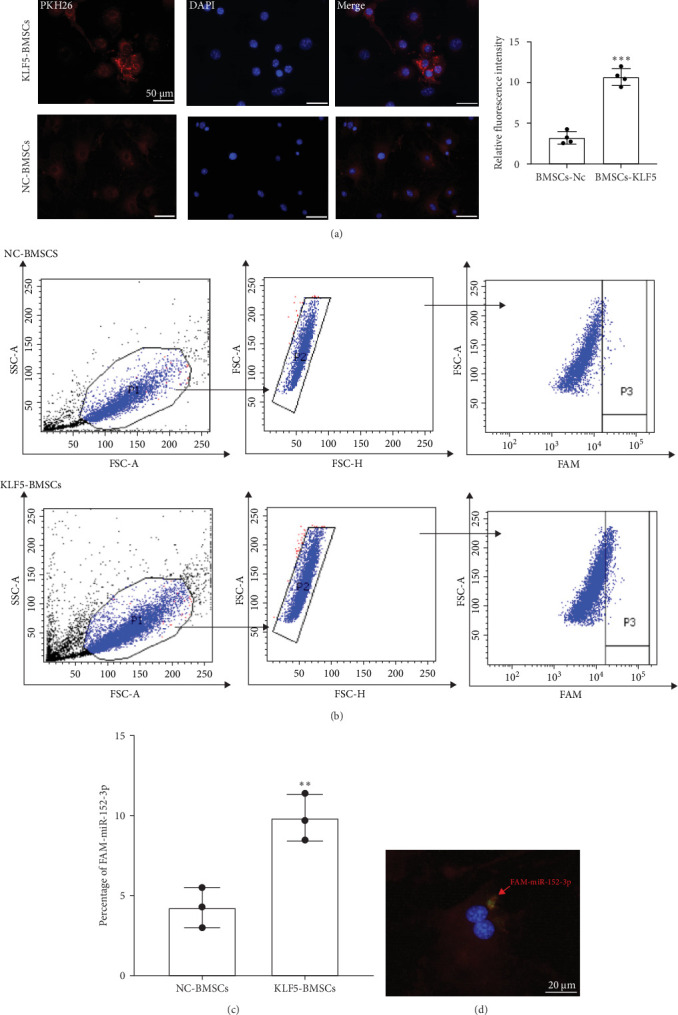
miR-152-3p in BMSCs induced by KLF5 can be transferred to CFs via exosome secretion. (A) Exosome were labeled with PKH26 red dye. After 24 hours of cocultured with CFs, they were fixed and imaged with a confocal microscope. (B) CFs were cocultured with FAM-miR-152-3p-transfected exosomes derived from NC-BMSCs and KLF5-BMSCs for 24 h. A flow cytometric analysis was used to determine the number of CFs containing FAM-152-3p. (C) And statistically analyzed. (D) The engulfment of FAM-miR-152-3p by CFs was captured with confocal microscopy. (*⁣*^*∗∗*^*p* < 0.01, *⁣*^*∗∗∗*^*p* < 0.001, as compared with NC-BMSCs group (N=3).).

RT-PCR was used to detect the expression levels of miR-152-3p in NC-BMSCs and KLF5-BMSCs as well as their extracellular vesicles. The results of the experiment, as shown in Figure 6, indicate that the expression of miR-152-3p is significantly higher in KLF5-BMSCs (NC-BMSCs vs. KLF5-BMSCs: 1.026 ± 0.041 vs. 2.169 ± 0.071, *p* < 0.001) and their extracellular vesicles (NC-BMSCs vs. KLF5-BMSCs: 1.05 ± 0.021 vs. 3.429 ± 0.048, *p* < 0.001). Based on all the results, we speculate that miR-152-3p secreted by BMSCs is regulated by KLF5. Considering KLF5 is a transcription factor, which may regulate the expression of miR-152-3p by binding to the potential sequence in its promoter region. We predicted the potential binding motifs of KLF5 within the sequence using JASPAR (http://jaspar.genereg.net/) and selected the binding site “gccccgcccc” with the highest relative score (Figure 7C). Dual luciferase reporter gene experiments showed that KLF5 enhance the luciferase activity driven by the Mir152 promoter (Figure 6D). Taken together, these results suggest that miR-152-3p is a direct downstream regulatory target of KLF5.

### 3.6. BMSCs-Derived miR-152-3p Is Transferred to CFs Through Exosomes

To investigate whether miR-152-3p transfer is mediated by the release of exosomes and whether CFs take up BMSCs-derived exosomes, we conducted coculture experiments using BMSCs and CFs. In these experiments, cells were separated by a membrane with 0.4 μm pore size to prevent direct cell-to-cell contact or transfer of larger vesicles. In this setup, BMSCs transfected with 5-Carboxyfluorescein (FAM)-labeled miR-152-3p were placed in the upper chamber, while CFs were seeded in the lower chamber. As shown in Figure 7, FAM-labeled-miR-152-3p in CFs was significantly increased following KLF5 overexpression. To further investigate the mechanism, whereby exosomes were taken up by CFs, we labeled BMSCs-derived exosomes with PKH26 and cocultured them with CFs. Confocal microscopy analysis revealed that CFs took up exosomes, particularly those derived from KLF5-transduced BMSCs. This finding was further supported by an increased level of miR-152-3p in CFs cocultured with pAd-KLF5-transduced BMSCs, once again indicating that BMSCs- derived exosomes can be taken up by CFs and that KLF5 promotes formation and transfer between BMSCs and CFs.

## 4. Discussion

Exosomes play a pivotal role as a conduit for the effects exerted by stem cells. Recent research suggests that stem cells can communicate by secreting or receiving miRNAs, which can then be transferred to other cells via exosomes [25]. In this study, our data illustrate that KLF5 exerts its anticardiac fibrosis effects by controlling the expression of miR-152-3p. We observed that miR-152-3p-rich exosomes were released from KLF5-overexpressing BMSCs and subsequently taken up by recipient CFs. miRNAs, LncRNAs, and other noncoding RNAs have also been confirmed to serve as pivotal regulators in canonical fibrotic pathways, including TGF-β and WNT pathway [26]. Prior studies have shown that miR-152-3p inhibits CFs proliferation and migration by suppressing the Wnt1/β-catenin pathway, resulting in a reduction in myocardial fibrosis [21]. Our findings are in line with these observations; compared to nonmodified control (NC) BMSCs, KLF5-BMSCs demonstrate an enhanced capacity to hinder CFs/CMFs transition, leading to decreased ventricular fibrosis and a mitigation of late VAs post-MI. Consequently, a therapeutic strategy involving the modulation of KLF5 or miR-152-3p in conjunction with stem cell therapy emerges as a promising and innovative approach for myocardial infarction (MI) treatment.

KLF5, a crucial transcription factor in cardiac remodeling, governs various vital biological processes [13, 26]. Previous studies have illustrated that activation of cardiomyocyte Klf5 limits doxorubicin-induced heart failure by mediating glucose metabolism [27]. Ma et al. [13] demonstrated that overexpression of KLF5 in vascular smooth muscle cells protected from vascular senescence and rupture of abdominal aortic aneurysm, in part due to reduced ROS. Notably, indirect regulation of KLF5 also contributes to cardiac pathophysiology. KLF5 transactivated IGF-1 in cardiac fibroblasts (CFs), and IGF-1 subsequently acted in a paracrine fashion to induce hypertrophic responses in cardiomyocytes [27–29]. In our investigation, we constructed BMSCs with heightened KLF5 expression. Compared to NC-BMSCs, the transplantation of KLF5-BMSCs for MI treatment resulted in reduced expression of α-SMA and collagen. Furthermore, KLF5-BMSCs therapy significantly diminished the vulnerability to VAs and sudden cardiac death post-MI. These outcomes substantiate our hypothesis that KLF5 enhances the reparative capacity of BMSCs following MI and modulates cardiac electrical activity.

Our study tested the effect of KLF5-BMSCs on CFs-to-CMFs transition via coculture with KLF5-BMSCs or NC-BMSCs. Our study demonstrated that KLF5-BMSCs inhibited CFs activation, leading to reduced collagen deposition and diminished expression of α-SMA under hypoxic conditions. Mias et al. [30] reported that BMSCs conditioned medium hindered the differentiation of CFs into CMFs, decreased CFs viability, and stimulated MMP2 and MMP9 activity in CFs, thereby significantly reducing collagen fiber deposition. Utilizing BMSCs conditioned medium in the process ignored the metabolic changes in BMSCs under hypoxic or other interventions. In our experiments, CFs were cocultured with BMSCs using cell culture inserts and subjected to hypoxic conditions. KLF5-BMSCs showed inhibitory effects on the differentiation of CFs-into-CMFs under hypoxia intervention through autocrine/paracrine actions rather than a mechanism of cell cell-to-cell connections. These results further support that the therapeutic benefits of BMSCs in MI treatment may predominantly stem from their paracrine and cytokine actions. We showed in vivo that KLF5-BMSCs can protect mice against MI. Compared to NC-BMSCs transplantation, KLF5-BMSCs transplantation reduced the amount of collagen I and sMA, thus, reducing the size of infarct areas and the extent of ventricular fibrosis.

The differentiation of CFs into CMFs not only triggers an increase in collagen I deposition, but also has an important impact on cardiac electrophysiological properties during the alteration of cardiac mechanical properties. Changing mechanical stress can activate intracellular signaling pathways like TGF-β, MAPK/ERK, and Wnt/β-catenin pathways, which in turn lead to changes in the expression of electrocoupling proteins, such as Cx43 and Cx45 thereby exacerbating cardiac electrical remodeling [26, 31]. This mechanism reveals the complex interaction between cardiac structure and electrical activity, emphasizing the critical role of CFs and CMFs in maintaining cardiac function and electrophysiological stability.

Conventional viewpoint asserts that the structural integrity and normal function of cardiomyocyte are fundamental for maintaining cardiac electrical activity, with CFs playing a key role in the cardiac structure [32]. The distribution of Cx43 and Cx45 in CMFs differs from that in CFs [33]. This variation is associated with changes in intercellular electrical coupling relationships within post-MI cardiac tissue. Under physiological conditions, CFs remain in a state of electrical quiescence and do not actively participate in cardiac electrical activity. In contrast, CMFs couple with cardiomyocytes, as well as with CFs or other CMFs, forming gap junctions that facilitate intercellular communication [34]. In this study, we found that hypoxia induced the transformation of CFs into CMFs, resulting in a significant increase in Cx43 expression. Coculturing with BMSCs significantly attenuated the hypoxia-induced increase in Cx43 expression, with KLF5 further enhancing the beneficial effects of BMSCs. Furthermore, previous studies have indicated that in rats, mice, dogs, and humans CFs possess resting membrane potentials [35] and are regulated by voltage-dependent potassium currents (Kv) and inward-rectifier potassium currents (Kir) [36]. An increase in Kir currents can lead to hyperpolarization of CFs and enhance calcium influx mediated by intracellular calcium stores, while an increase in Kv currents can enhance CFs proliferative capacity. Our study revealed that hypoxia led to significant decreases in Kv4.2 and Kv1.2 and significant increases in Kir2.1, Kir6.1, and Kir2.3 in CFs. This indicates that hypoxia promotes CFs proliferation and hyperpolarization. Coculturing with NC-BMSCs and KLF5-BMSCs suppressed the abnormal changes in Kv and Kir induced by hypoxia, with KLF5-BMSCs exhibiting more pronounced effects.

The present study has certain limitations that warrant further discussion. For instance, membrane patch-clamp techniques were employed to detect changes in Kir and Kv currents in CFs, while mapping techniques were utilized to detect alterations in cardiac electrical conduction. However, the involvement of other chemotactic factors, growth factors, and miRNAs cannot be ruled out, future investigations will need to focus on elucidating their roles in cardiac function and arrhythmia following MI.

## 5. Conclusion

In this study, our results demonstrate that the overexpression of KLF5 enhances the ability of BMSCs to decrease the frequency and susceptibility of VAs in MI mice. These effects are linked to KLF5′s function in upregulating the expression of miR-152-3p in BMSCs, which is then conveyed to CFs via exosomes. Consequently, this cascade of events leads to the inhibition of the transformation of CFs into CMFs.

## Figures and Tables

**Table 1:** Heart function after MI in mice.

Parameter	Sham	MI	NC-BMSCs	KLF5-BMSCs
LVEF (%)	63.96 ± 2.37	26.13 ± 1.38	35.64 ± 2.61	34.48 ± 1.89
LVFS (%)	34.11 ± 1.04	12.35 ± 0.30	16.39 ± 0.92	16.26 ± 0.76
LVIDd (mm)	3.18 ± 0.15	3.61 ± 0.11	3.52 ± 0.19	3.60 ± 0.12
LVIDs (mm)	2.09 ± 0.07	3.16 ± 0.10	2.94 ± 0.16	3.02 ± 0.12
IVSs (mm)	1.18 ± 0.16	0.65 ± 0.02	0.72 ± 0.01	0.71 ± 0.04
IVSd (mm)	0.78 ± 0.019	0.49 ± 0.019	0.57 ± 0.02	0.55 ± 0.03

Abbreviations: IVSd, interventricular septal thickness at end diastole; IVSs, interventricular septal thickness at end systole; LVEF, left ventricular ejection fraction; LVFS, left ventricular fractional shortening; LVIDd, left ventricular internal diameter at end-systole; LVIDs, left ventricular internal dimension systole.

**Table 2:** Scoring criteria.

Without PVC, VT, VF	PVC	VT < 5	VT ≥ 5	VF (2–5)	VF > 5
0	1	2	3	4	5

Abbreviations: PVC, premature ventricular contraction; VF, ventricular fibrillation; VT, ventricular tachycardia.

**Table 3:** Electrocardiogram monitoring post-MI in mice.

Parameter	Sham	MI	NC-BMSCs	KLF5-BMSCs
HR (n/min)	572.6 ± 21.3	574.9 ± 14.6	570.6 ± 19.5	566.3 ± 25.9
PVC (n/h)	2.8 ± 0.9	55.3 ± 6.4^†††^	39.3 ± 5.1*⁣*^*∗∗∗*^	28.4 ± 6.1*⁣*^*∗∗∗*^,##
VB (n/h)	1.1 ± 0.6	12.6 ± 2.7^†††^	9.4 ± 1.9*⁣*^*∗∗*^	5.4 ± 1.3*⁣*^*∗∗∗*^,###
VT1 (n/h)	0.088 ± 0.1	1.5 ± 0.5^†††^	1.3 ± 0.3	1.1 ± 0.4*⁣*^*∗*^
VT2 (n/h)	0.1 ± 0.2	7.9 ± 1.6^†††^	6.7 ± 1.3	4.4 ± 1.2*⁣*^*∗∗∗*^,##

*Note*: ^†††^*p* < 0.001, as compared with control group; *⁣*^*∗*^*p* < 0.05, *⁣*^*∗∗*^*p* < 0.01, *⁣*^*∗∗∗*^*p* < 0.001, as compared with MI group; ^#^*p* < 0.05, ^##^*p* < 0.01, ^###^*p* < 0.001, as compared between NC-BMSCs and KLF5-BMSCs group.

Abbreviations: HR, heart rhythm; PVC, premature ventricular contraction; VB, ventricular bigeminy; VT1, ventricular trigeminy; VT2, ventricular tachycardia.

**Table 4:** VA score in mice after MI.

VA scores	Sham	MI	NC-BMSCs	KLF5-BMSCs
0	7 (10)	0 (10)	2 (10)	4 (10)
1	2 (10)	2 (10)	4 (10)	4 (10)
2	1 (10)	2 (10)	1 (10)	2 (10)
3	0 (10)	3 (10)	1 (10)	1 (10)
4	0 (10)	2 (10)	1 (10)	0 (10)
5	0 (10)	1 (10)	1 (10)	0 (10)
Mean ± SD	0.4 ± 0.7	2.8 ± 1.3^†††^	1.8 ± 1.7*⁣*^*∗∗*^	1 ± 1.1*⁣*^*∗∗∗*^,#

*Note*: ^†††^*p* < 0.001, as compared with control group; *⁣*^*∗*^*p* < 0.05, *⁣*^*∗∗*^*p* < 0.01, *⁣*^*∗∗∗*^*p* < 0.001, as compared with MI group; ^#^*p* < 0.05, ^##^*p* < 0.01, ^###^*p* < 0.001, as compared between NC-BMSCs and KLF5-BMSCs group.

Abbreviation: VAs scores, ventricular arrhythmias scores.

**Table 5:** Relative electronic ion channels mRNA expressions in each groups.

Ion channel	Control	Hypoxia	NC-BMSCs	KLF5-BMSCs
Kv1.2	1.009 ± 0.002	0.322 ± 0.04^†††^	0.717 ± 0.022*⁣*^*∗∗∗*^	0.774 ± 0.037*⁣*^*∗∗∗*^
Kv4.2	1.035 ± 0.043	0.217 ± 0.042^†††^	0.624 ± 0.036*⁣*^*∗∗∗*^	0.753 ± 0.027*⁣*^*∗∗∗*^^,#^
Kir2.1	1.00 ± 0.025	2.065 ± 0.096^†††^	1.637 ± 0.057*⁣*^*∗∗∗*^	1.249 ± 0.035*⁣*^*∗∗∗*^^,##^
Kir2.3	1.02 ± 0.046	2.103 ± 0.168^†††^	1.548 ± 0.055*⁣*^*∗∗∗*^	1.303 ± 0.079*⁣*^*∗∗∗*^
Kir6.1	1.02 ± 0.038	1.78 ± 0.025^†††^	1.602 ± 0.064*⁣*^*∗∗*^	1.391 ± 0.055*⁣*^*∗∗∗*^^,#^

*Note*: ^†††^*p* < 0.001, as compared with control group; *⁣*^*∗*^*p* < 0.05, *⁣*^*∗∗*^*p* < 0.01, *⁣*^*∗∗∗*^*p* < 0.001, as compared with Hypoxia group; ^#^*p* < 0.05, ^##^*p* < 0.01, ^###^*p* < 0.001, as compared between NC-BMSCs and KLF5-BMSCs group.

## Data Availability

The data that support the findings of this study are available upon request from the corresponding author. The data are not publicly available due to privacy or ethical restrictions.

## Supporting Information

Supporting Information 1 Figure S1: Identification of BMSCs; Figure S2: Expression of KLF5 detected by qRT-PCR after transfection; Figure S3: Identification of Exosome; Figure S4: Schematic representation of the coculture experiment.

Supporting Information 2 Supporting Information 2: The sequences of the Mir152 promoter.
