# Supplementary material for: KLF5 Regulation of Exosome-Derived miR-152-3p From Bone Marrow Stem Cells Improves Ventricular Arrhythmia After Myocardial Infarction
Source: Stem Cells Int. 2025 Aug 9;2025:5572221. doi: 10.1155/sci/5572221 (PMC12357777; doi:10.1155/sci/5572221)
Supplement: Supporting Information 1 — Figure S1: Identification of BMSCs; Figure S2: Expression of KLF5 detected by qRT-PCR after transfection; Figure S3: Identification of Exosome; Figure S4: Schematic representation of the coculture experiment. [file 5572221.f1.docx]

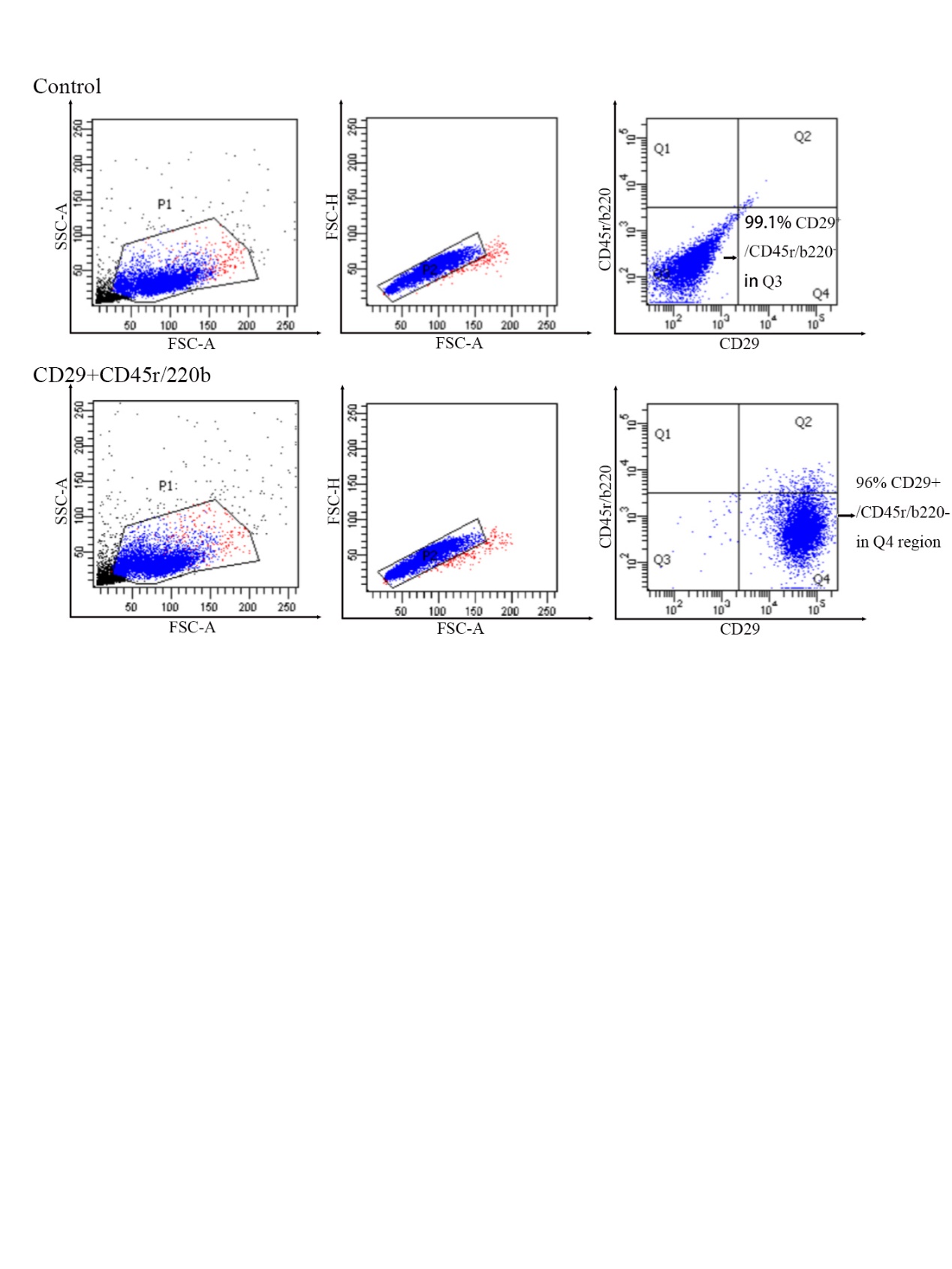


**Supplemental Figure 1. Identification of BMSCs**

Flow cytometry analysis showed that about 96.5% of cells were positive for CD29 surface markers, but CD45r/b220 were negative (0.6%). Accordingly, the cells employed in this study were recognized as BMSCs

**Supplemental Figure 2. Expression of KLF5 detected by qRT-PCR after transfection**

Gene expression levels of KLF5 by RT-PCR in NC-BMSCs and KLF5-BMSCs group (^***^P<0.001, as compared between NC-BMSCs and KLF5-BMSCs group)


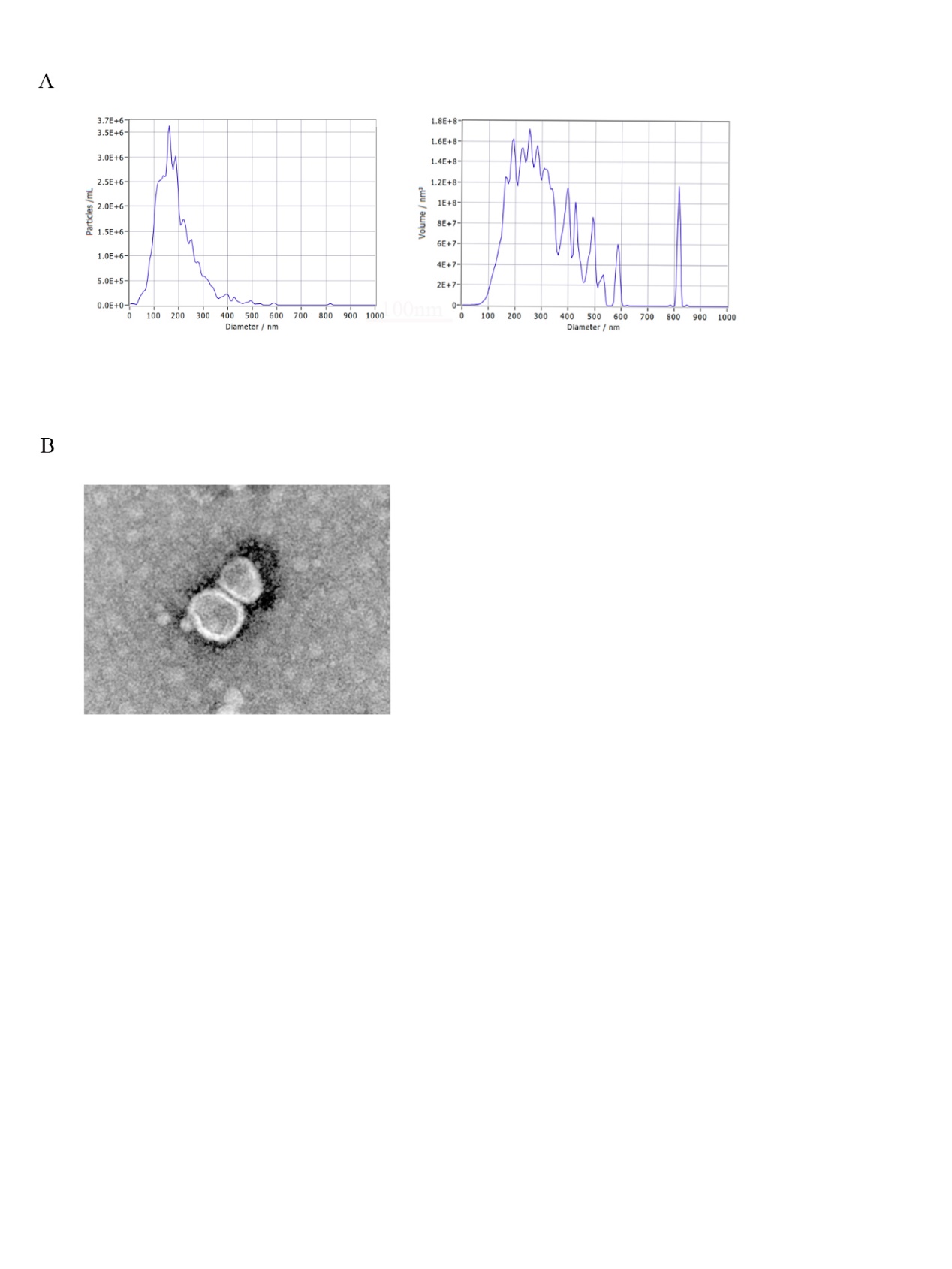


**Supplemental Figure 3. Identification of Exosome**

(A) Exosome particle size and concentration examined through NTA. (B) Electron microscopy of negative-stained exosome.


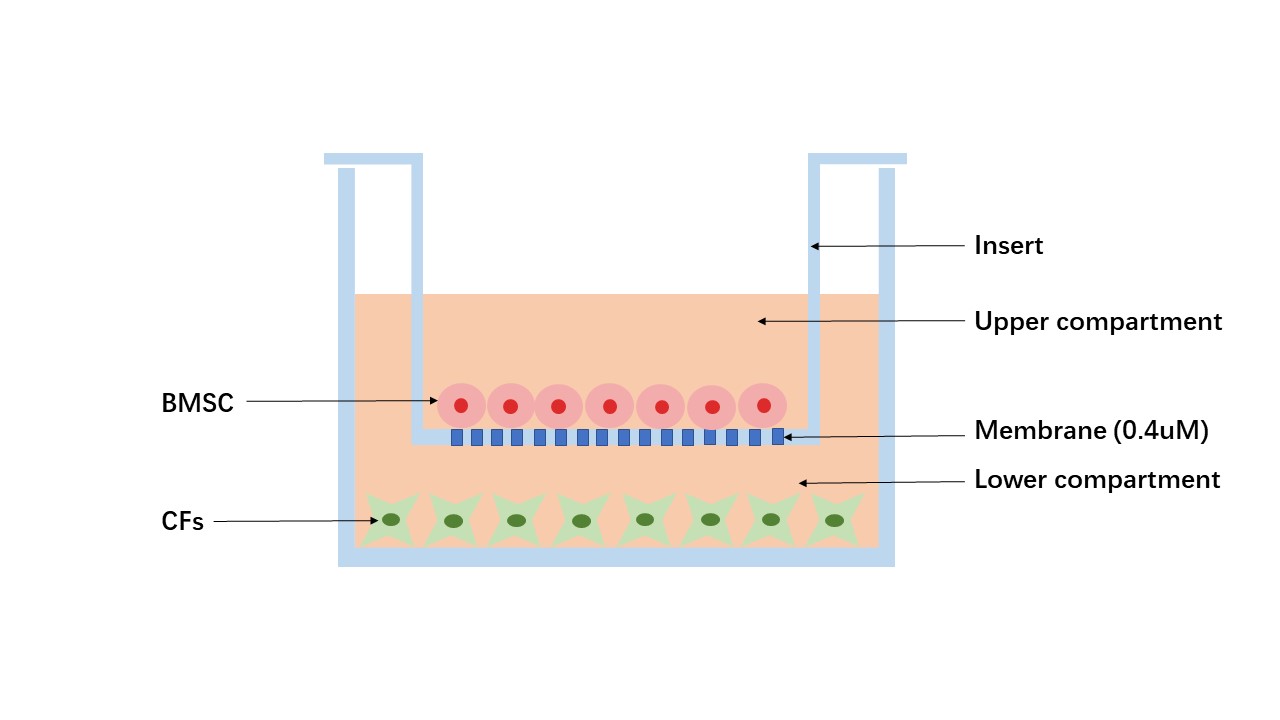


**Supplemental Figure 4. Schematic representation of the co-culture experiment.**
