# Supplementary material for: KLF5 Regulation of Exosome-Derived miR-152-3p From Bone Marrow Stem Cells Improves Ventricular Arrhythmia After Myocardial Infarction
Source: Stem Cells Int. 2025 Aug 9;2025:5572221. doi: 10.1155/sci/5572221 (PMC12357777; doi:10.1155/sci/5572221)
Supplement: Supporting Information 2 — The sequences of the Mir152 promoter. [file 5572221.f2.docx]

pGL3-Mir52-promoter containing the -1926 bp to +73 bp promoter sequence of Mir152 and the highlighted part are binding sites.

WT Sequence

CGGTAGATGGAGAGAAGTGACTCCTTTAAGCTGTCCTCTGATCGCCACGTGAGCCCTGTGTCACACCCTCCCCTCATCAATAATTACTAAGTAAAATAAAAGCGTAAACAAAAGGATCAGTAAGAGTAATTGTTAAAAAACAAAAAAAGGTGAAAGTTGGAAAGAGAAGTCTTAAGCAGAAGGAAGTCTGGTGTGAGCGACTGTGATGTTCTACACTGAGGCTGTCAAGGGCAGAATGGGAAAACCCGTGGAACCCTCCTACCCAACTAATTAGCCCAACCAGCTTGAAAAGGTCACACGAAGTCTGACTCCTCTCAATGGGTATGTCTGAAAACACCTCCCTAGGCACGGTGACTTCTGACATACAATGAGAGCGCTGGCTCGAGTATAAGACACATCTGTCAGTGTTGTGAATGGCTGGCTGCCCAAGCACCAGTAACTACCTCACTGTATGTGGTCATACTCTGGCCGGGTTAGTCCACAGGCTCAAGCTGGACTTCAGGTGTGTTGTGACCCTACTGGCTTCATCCACCTCTGGGTCTTGAAAAATGTCTGGCGGAGGTGTCCTTCAGCCCATGGTTCTTTTCCCCAGGTACTATGAGGGGAAGCGGGGAACTCTTCCGAGTGGAGAGGTCTCACTGACAGGCTCTGTCTTGAGATCAAAAAGGTGACTTGAGGAGCTGGAGAGATGGCTCAGTGGTTAAGAGCCCTGGCTGCTCTTCCAGAGGTCCTGAATTCTATTCCCAGCAACCACATGGTGGCTCACAACTATCTATAATGGGATCTGATGCCCTCTTCTGGTGTGTCTGAAGACAGCGACAGTGTACTCACATTAAAAAAAAAAAAAAAATCTCAAAAATTAAAAAAAAGAGAGTTCTTCAGCACTCGGGAGGCAGAGGGAGGCGGATTTCTGAGTTCGAGGCCAGCTTGGTCTACAAAGTGAGTTCCAGGACAGCCAGGGCTGTATAGAGAAACCTTGACTCGAAAAACCAAAAAAAAAAAAAAAAGAGAGAGAGAGAGAGAGTTCTTTTTTAAAAAAGTGACTTGAACTATTTGTTCCAATGATACCTACTAGGAGGTCCTGAGGACAAAGTGAACCTAAACTGAAATTTGGGCTGTCCCAGAACAGCCACATGGAGACCTCTGTGCACCAAAGCCACAGGCGCCCTAGGCCAGAAGAGGCAGTAAGTGGGAAGAAAAGAGTAGGCTCTGGTTCACCTGAAAGACTGGGAGGTGCACAGAGGAGCCCAGAGGGAAGAATCCAAGTAGGGAGACGAGGTTCTGCAACGACGTGGGGGTCGGGCAGAAAGCTCGCCCAGAGAGAGAGGACGCACACAGAGGCCAGCCACGCAGGACTGGGCGGAGGGGCGGCGTCGCGGGGGGCGGGCACGGGGCGGGGCGGCGCTGTCAGTGCAGGCAGCGAGCGGAATGCAGCGGCCGGAGGCCTGGCCACGTCCGCACCCGGGGGAGGGGGCCTCAGCCGCCCAAGCCGGGGGCGCAGCGCCGCCCACCCGAGCCACGGAACAGCGGGTAGGGAGCACGGAGTGTGGGGTCCAGGGGCAGCAGGATCGGTGGTCGGGGGCAACTGATTTACCTCGGGCTTCTAAGCTGGGAACTTTGTGCCACCACTGCCCAGCCCCGGAGAGGGAGCCCGGGCCCAGGAAGGGGAGAAAAAGTTTGTCCTGGAGCGCACGGGCGAGGAGGCCTTGCCTGAGTGGAGTGTCGCAGGAGGGGCTGAAGTTCTGGGGAACTCGCCGCAGTTATAACCGACTTGGGTGGCCGGGATGGAAGCGAGATTCTGGCGGGACGAGGGAGGAGTGGGAGTGGCGAGGTGGTCTGGTTGGGTTCCCTTGCTCCAGAATGATGGCCGCGCGCATCTGGAACTGGGGGTCAGCTGGACATAAGGAGGCTCGTCGCCGCTGTTCCCCGGGCCTAGGTTCTGTGATACACTCCGACTCGGGCTCTGGAGCAGTCAGTGCATGACAGAACTTGGGCCCGG

Mutant Sequence

CGGTAGATGGAGAGAAGTGACTCCTTTAAGCTGTCCTCTGATCGCCACGTGAGCCCTGTGTCACACCCTCCCCTCATCAATAATTACTAAGTAAAATAAAAGCGTAAACAAAAGGATCAGTAAGAGTAATTGTTAAAAAACAAAAAAAGGTGAAAGTTGGAAAGAGAAGTCTTAAGCAGAAGGAAGTCTGGTGTGAGCGACTGTGATGTTCTACACTGAGGCTGTCAAGGGCAGAATGGGAAAACCCGTGGAACCCTCCTACCCAACTAATTAGCCCAACCAGCTTGAAAAGGTCACACGAAGTCTGACTCCTCTCAATGGGTATGTCTGAAAACACCTCCCTAGGCACGGTGACTTCTGACATACAATGAGAGCGCTGGCTCGAGTATAAGACACATCTGTCAGTGTTGTGAATGGCTGGCTGCCCAAGCACCAGTAACTACCTCACTGTATGTGGTCATACTCTGGCCGGGTTAGTCCACAGGCTCAAGCTGGACTTCAGGTGTGTTGTGACCCTACTGGCTTCATCCACCTCTGGGTCTTGAAAAATGTCTGGCGGAGGTGTCCTTCAGCCCATGGTTCTTTTCCCCAGGTACTATGAGGGGAAGCGGGGAACTCTTCCGAGTGGAGAGGTCTCACTGACAGGCTCTGTCTTGAGATCAAAAAGGTGACTTGAGGAGCTGGAGAGATGGCTCAGTGGTTAAGAGCCCTGGCTGCTCTTCCAGAGGTCCTGAATTCTATTCCCAGCAACCACATGGTGGCTCACAACTATCTATAATGGGATCTGATGCCCTCTTCTGGTGTGTCTGAAGACAGCGACAGTGTACTCACATTAAAAAAAAAAAAAAAATCTCAAAAATTAAAAAAAAGAGAGTTCTTCAGCACTCGGGAGGCAGAGGGAGGCGGATTTCTGAGTTCGAGGCCAGCTTGGTCTACAAAGTGAGTTCCAGGACAGCCAGGGCTGTATAGAGAAACCTTGACTCGAAAAACCAAAAAAAAAAAAAAAAGAGAGAGAGAGAGAGAGTTCTTTTTTAAAAAAGTGACTTGAACTATTTGTTCCAATGATACCTACTAGGAGGTCCTGAGGACAAAGTGAACCTAAACTGAAATTTGGGCTGTCCCAGAACAGCCACATGGAGACCTCTGTGCACCAAAGCCACAGGCGCCCTAGGCCAGAAGAGGCAGTAAGTGGGAAGAAAAGAGTAGGCTCTGGTTCACCTGAAAGACTGGGAGGTGCACAGAGGAGCCCAGAGGGAAGAATCCAAGTAGGGAGACGAGGTTCTGCAACGACGTGGGGGTCGGGCAGAAAGCTCGCCCAGAGAGAGAGGACGCACACAGAGGCCAGCCACGCAGGACTGGGCGGAGGGGCGGCGTCGCGGGGGGCGGGCACTTTTATTTTAGGCGCTGTCAGTGCAGGCAGCGAGCGGAATGCAGCGGCCGGAGGCCTGGCCACGTCCGCACCCGGGGGAGGGGGCCTCAGCCGCCCAAGCCGGGGGCGCAGCGCCGCCCACCCGAGCCACGGAACAGCGGGTAGGGAGCACGGAGTGTGGGGTCCAGGGGCAGCAGGATCGGTGGTCGGGGGCAACTGATTTACCTCGGGCTTCTAAGCTGGGAACTTTGTGCCACCACTGCCCAGCCCCGGAGAGGGAGCCCGGGCCCAGGAAGGGGAGAAAAAGTTTGTCCTGGAGCGCACGGGCGAGGAGGCCTTGCCTGAGTGGAGTGTCGCAGGAGGGGCTGAAGTTCTGGGGAACTCGCCGCAGTTATAACCGACTTGGGTGGCCGGGATGGAAGCGAGATTCTGGCGGGACGAGGGAGGAGTGGGAGTGGCGAGGTGGTCTGGTTGGGTTCCCTTGCTCCAGAATGATGGCCGCGCGCATCTGGAACTGGGGGTCAGCTGGACATAAGGAGGCTCGTCGCCGCTGTTCCCCGGGCCTAGGTTCTGTGATACACTCCGACTCGGGCTCTGGAGCAGTCAGTGCATGACAGAACTTGGGCCCGG
